# Revealing Mechanopathology Induced by Dengue NS1 Using Organ Chips and Single-Cell Force Spectroscopy

**DOI:** 10.1021/acsbiomaterials.4c02410

**Published:** 2025-03-25

**Authors:** Huaqi Tang, Tom M. J. Evers, Mehrad Babaei, Alireza Mashaghi

**Affiliations:** Medical Systems Biophysics and Bioengineering, Leiden Academic Centre for Drug Research, Faculty of Science, Leiden University, 2334CC Leiden, The Netherlands

**Keywords:** dengue, organ chip, single-cell rheology, acoustic force spectroscopy, mechanopathology, climate change

## Abstract

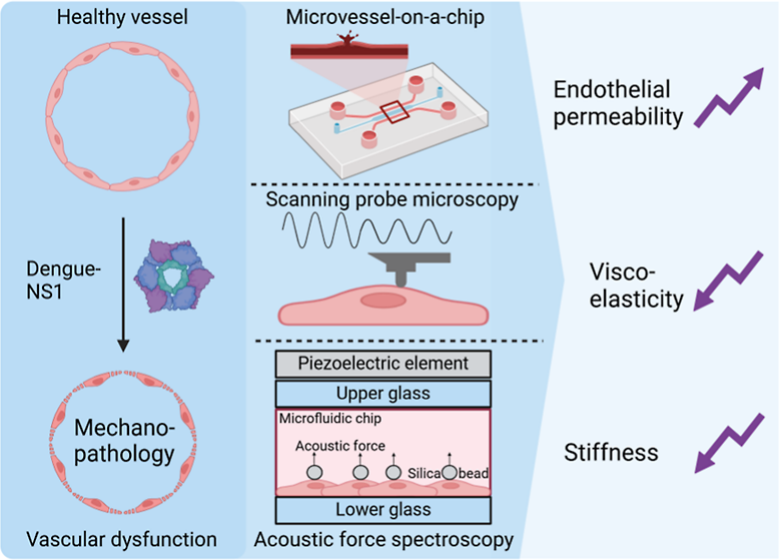

Capillary leakage is a hallmark of severe dengue, yet its precise mechanisms remain elusive. Emerging evidence highlights the dengue virus’s targeting of mechanically active endothelial cells as a key contributor to dengue shock syndrome. The viral nonstructural protein 1 (NS1) has been identified as a central player, disrupting endothelial integrity and inducing vascular hyperpermeability independently of pro-inflammatory cytokines. This study provides a direct assessment of NS1-induced endothelial pathology by combining single-cell force spectroscopy and a microvessel-on-a-chip platform. We demonstrate that NS1 significantly alters endothelial cell mechanics, reducing cell stiffness and compromising junctional integrity, thereby directly linking these mechanical alterations to vascular dysfunction. These findings establish a framework for understanding the mechano-pathology of dengue and offer a platform for developing targeted therapeutic strategies to mitigate severe disease outcomes.

## Introduction

Dengue fever is a mosquito-borne viral illness plaguing tropical and subtropical regions. With an estimated 100–400 million infections annually, it poses a significant health risk.^1^ Approximately half a million people develop a critical form known as dengue shock syndrome (DSS), where a dramatic drop in blood pressure due to fluid leakage can be fatal. Currently, there is no cure or specific antiviral treatment for dengue. Symptoms range from fever, body aches, and rashes to complications like prolonged hair loss. Endemic in areas with *Aedes* mosquitos, its prevalence is expected to rise due to urbanization and climate change, earning it the label of a “disease of the future.”^2−4^ With 50% of the world’s population at risk and an economic burden exceeding $8.9 billion in 2013 alone,^5,6^ understanding the disease is crucial for developing effective treatments. Vaccine development has been challenging, with the only FDA-approved option, Dengvaxia, having limited effectiveness and utility restrictions. In fact, for children without prior dengue infection, the vaccine can increase the risk of severe illness upon future infection.^7,8^

Dengue fever presents a unique challenge in our comprehension of infectious diseases. While capillary leakage, the hallmark symptom, is readily observed, the underlying mechanisms remain poorly understood. Recent research proposes a compelling model: dengue as a “disease of mechanics.” This model posits that the virus targets mechanically active cells and structures including the endothelial barrier, leading to fatal shock. Although cytokines and vasoactive agents released by Dengue-infected cells likely increase vascular permeability, viral proteins may also directly disrupt endothelial monolayer integrity. Dengue virus nonstructural protein 1 (NS1) is the only viral protein secreted by infected host cells, with high circulating levels observed in severe dengue patients, correlating with the development of DSS.^9,10^ Previous animal studies suggest that NS1 can directly trigger vascular leakage in the absence of infection.^11−13^ In vitro studies using *trans*-endothelial electrical resistance indicate that NS1 can induce endothelial hyperpermeability in 2D endothelial monolayers without the presence of cytokines such as TNF, IL-6, and IL-8.^13^

The mechanical properties of endothelial cells, including their softness or stiffness, are key indicators of cellular function and integrity.^14^ Softness refers to the ability of a cell to deform under mechanical stress, while stiffness describes its resistance to deformation.^15^ Both properties are crucial for maintaining the structural integrity of the endothelium, which functions as a selective barrier to regulate vascular permeability. The mechanical behavior of endothelial cells combines both elastic and viscous properties, meaning they exhibit both rigidity and the ability to dissipate energy over time. This viscoelasticity is essential for the dynamic regulation of endothelial function, allowing cells to respond to mechanical forces like shear stress, and to adapt to changes in their environment. Disruptions in endothelial softness, stiffness, or viscoelasticity, can impair the ability of endothelial cells to maintain their barrier function, leading to increased permeability and vascular dysfunction. By examining these mechanical properties in response to NS1 exposure, one can gain insights into how alterations in endothelial mechanics contribute to the pathogenesis of dengue and other vascular diseases.

To investigate NS1-induced mechanical alterations, we employ an integrated approach combining advanced single-cell force spectroscopy techniques acoustic force spectroscopy (AFS) and scanning probe microscopy (SPM), with a microvessel-on-a-chip platform. AFS enables high-throughput force measurements across populations of endothelial cells, while SPM provides nanoscale resolution and real-time mechanical characterization at the single-cell level.^16−20^ Together, these techniques provide a comprehensive view of endothelial mechanics, capturing both collective and single-cell behavior. Complementing these, our microvessel-on-a-chip system models vascular integrity under physiologically relevant flow conditions, bridging the gap between in vitro and in vivo studies.^21,22^

Using this integrated approach, we directly examine the impact of NS1 on endothelial cell mechanics and vascular integrity. Our findings demonstrate that NS1 significantly alters cellular mechanics at the single-cell level and disrupts endothelial junction integrity, consequently affecting the junctional mechanics of the endothelial lining. By uncovering these mechano-pathological changes, our study provides critical insights into how dengue fever compromises vascular function. These discoveries pave the way for novel research avenues and may inform the development of targeted therapeutic strategies to mitigate dengue-induced vascular dysfunction.

## Materials and Methods

### Cell Culture

Human umbilical vein endothelial cells (HUVECs) were prepared according to established protocols from Leiden University Medical Center. Prior to cell seeding, flasks were coated with 2% gelatin solution (G1393, Sigma-Aldrich) and incubated for 30 min at 37 °C with 5% CO_2_ to promote cell adhesion. The gelatin solution was then removed, and Endothelial Cell Growth Medium 2 (ECM2; C-22011; PromoCell), supplemented with growth factors, was added. HUVECs were seeded into the prepared flasks and maintained at 37 °C with 5% CO_2_. The medium was changed every 48 h to ensure optimal cell health.

### Microvessel-on-a-chip Preparation

The OrganoPlate 2-lane 96 (9605-400-B; Mimetas) was used to prepare the microvessel-on-a-chip. A 3D Culture Matrix Rat Collagen I (3447-020-01; R&D Systems), neutralized to a final concentration of 4 mg/mL using 10% 37 g/L Na_2_CO_3_ (S5761; Sigma) and 10% 1 M HEPES buffer (15630-056; Gibco), was added to the extracellular matrix (ECM) channels and polymerized at 37 °C with 5% CO_2_ for 10 min. To ensure optical clarity and to prevent gel dehydration, 50 μL of Hanks’ balanced salt solution (HBSS, 14025050; Gibco) was added to the observation windows. Before cell seeding, the microvascular channels were coated with a gelatin solution for 30 min. HUVECs were then seeded at 15 × 10^6^ cells/ml into the gelatin-coated microvascular channels and incubated for 1 h to facilitate microvessel formation. Following, 100 μL of cell culture medium was added, and the device was placed on an interval rocker platform with a 7° inclination and an 8 min cycle time to maintain continuous perfusion. The medium was refreshed after 24 h, and HUVECs were cultured for an additional 3–4 days.

### Vascular Permeability Assay

Microvascular channels were exposed to Dengue Virus Serotype 2 NS1 Protein (DENV2-NS1; The Native Antigen Company) at concentrations of 1, 5, 10, and 20 μg/mL, and incubated for 4 h. After refreshing the ECM channels with 20 μL of HBSS, the medium in the inlets and outlets of the microvascular channels was replaced with 40 μL and 30 μL of 125 μg/mL Alexa Fluor 555-conjugated albumin (A34786; Invitrogen), respectively. Time-lapse images were captured at 1 min intervals for 10 min using a fluorescence microscope (Nikon Eclipse Ti) with an environmental chamber (5% CO_2_, 37 °C).

The permeability coefficient was calculated by determining the fluorescence intensities in the microvascular (*I*_Vessel_) and ECM (*I*_ECM_) channels at each time point. The apparent permeability (*P*_app_) was calculated as
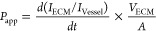
1where *I*_ECM_/*I*_Vessel_ is the ratio of intensity between the ECM and microvascular channel, *V*_ECM_ is the volume of the ECM channel (4.7 × 10^–4^ cm^3^), *A* is the surface area of the vessel wall between the ECM and microvascular regions (2.12 × 10^–2^ cm^2^). The resulting scatter plot was fitted with a linear trend line to determine the slope, which indicated the change in the intensity ratio inside the ECM channels as a function of time.

### Immunohistochemistry

The culture medium was aspirated from the microvessel channels, and cells were fixed with 4% paraformaldehyde (PFA, 28908; Thermo Scientific) for 10 min at room temperature, followed by rinsing with HBSS. VE-cadherin and actin filaments were detected after permeabilization with 0.2% Triton X-100 (T8787; Sigma-Aldrich) for 2 min, and blocking with 5% bovine serum albumin (BSA, A9647; Sigma-Aldrich) for 30 min at room temperature, and incubated with the primary antibody solution overnight at 4 °C. Mouse antihuman CD144 (1:100, 555661; BD Pharmingen) was used as the primary antibody, followed by Hoechst (1:2000, H3569; Invitrogen), Phalloidin–Tetramethylrhodamine B isothiocyanate (1:200, P1951; Sigma-Aldrich), and Alexa Fluor 488-conjugated goat antimouse secondary antibody (1:250, R37120; Invitrogen). Quantification of Pearson correlation coefficient for the colocalization of VE-cadherin and F-actin was performed using ImageJ colocalization analysis plugin Coloc2. Results are depicted as mean ± SEM (*n* = 5).

For hyaluronan immunostaining, nonspecific binding was blocked with an Endogenous Biotin-Blocking Kit (E21390; Invitrogen) and 5% BSA. After washing, cells were incubated overnight with biotinylated hyaluronan-binding protein (1:100, AMS.HKD-BC41; AMSBIO). Alexa Fluor 488 streptavidin conjugate (1:1000, S32354; Invitrogen) and Hoechst were used to detect hyaluronan and DAPI, respectively. Images of the stained cells were obtained using a high-content confocal microscope (ImageXpress Micro Confocal; Molecular Devices) and processed with ImageJ.

### Scanning Probe Microscopy

Experiments were performed using a CellHesion 200 scanning probe microscope (SPM) (Bruker, Germany), equipped with a Eurotherm 2216e heating element to maintain the experimental temperature at a physiological 37 °C. Sample dishes were placed under the SPM head, which was gradually lowered until the cantilever was submerged in the medium. Prior to each experiment, the thermal equilibration of the cantilever was confirmed by monitoring its deflection until fluctuations stabilized below 0.2 V.

We used SAA-SPH-5UM cantilevers (Bruker, Germany) made of Si_3_N_4_ with a hemispherical tip (23 μm height, 5 μm radius). To minimize nonspecific adhesion between the cantilever tip and the cell surface, cantilevers were coated with 1% Pluronic solution for 30 min at room temperature. Amplitude calibration was conducted using the thermal noise method. The SPM system was coupled to an Eclipse Ti2 phase-contrast microscope (Nikon, Japan) equipped with a 20*X*/0.4 objective to provide real-time in situ imaging of the experiments. Cells were selected based on micrographs, and the cantilever was positioned with a landing velocity of 5 μm/s. An indentation force of 0.4 ± 0.1 nN was applied. Upon reaching the preset force, the cantilever height was held constant for a 2-s pause period to allow the deflection to stabilize. Oscillatory indentation was then performed, with a 10 nm amplitude and frequencies ranging from 1 to 100 Hz (seven frequencies per decade, spaced logarithmically). During this process, the cantilever height and deflection signals were recorded. After measurements were completed, the probe was retracted at a velocity of 5 μm/s. This procedure was repeated for each frequency individually.

The CellHesion evaluation software (version 8.0.159) was used to compute the complex shear modulus (G*) of the cells. Oscillatory head height *h*(*t*) and force deflection *F*(*t*) data were fitted to sinusoidal models
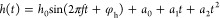
2and
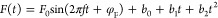
3here, *h*_0_ and *F*_0_ represent oscillation amplitudes, *f* is the frequency, and φ_h_ and φ_F_ are the phase angles. Polynomial terms (*a*_0_, *a*_1_, *a*_2_, *b*_0_, *b*_1_, *b*_2_) account for baseline drifts during measurements. The phase difference Δφ was determined as Δφ = φ_F_ – φ_h_.

The complex shear modulus can be calculated as
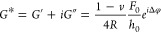
4where *R* is the radius of the indenter tip, and ν (Poisson’s ratio) is assumed to be 0.5 for cells. The real (storage) and imaginary (loss) components of the modulus were computed as
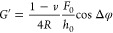
5and
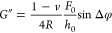
6

The loss tangent was defined as 

Hydrodynamic drag, which causes phase lag due to viscous friction, was accounted for using the flat amplitude-phase calibration method. The indenter probe was positioned on the hard polystyrene surface of the dish, and oscillations with a 2 nm amplitude were applied. On a hard surface, identical oscillations in head height and cantilever deflection are expected. The measured phase lag φ_l_ was modeled as
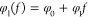
7the frequency-dependent phase differences (Δφ) were corrected based on this hydrodynamic drag calibration.

### Acoustic Force Spectroscopy Setup and Specifications

Details on the setup, specifications, and fabrication of the AFS have been previously described by our group.^18,19^ Briefly, all measurements were conducted on an AFS-G2 (Lumicks B.V., The Netherlands), which comprises a motorized z-stage mounted on an inverted microscope (Nikon Eclipse TE200), G2 AFS chip holders (microfluidic devices), and a temperature controller. Visualization and illumination were achieved with a motorized 20× microscope objective for nanometer-precise z-translation, a red-light LED, and a uEye camera (UI-324xCP-M, IDS) capable of imaging at a sampling frequency of 60 Hz. The AFS chip consists of a microfluidic chamber for cell loading and a piezoelectric element on top to transduce acoustic waves. Acoustic forces are applied to silica microbeads (7.9 μm in diameter; SIP-60–10, Spherotech), which were tracked by image recording using LabVIEW (provided by Lumicks B.V.) for real-time *x*, *y*, and *z* tracking of bead location. The resonance frequency used for all experiments was 14.47 MHz.

### Loading of the AFS Microfluidic Chamber

To allow HUVEC spreading, the microfluidic chamber of the AFS chip was first coated with gelatin. A cell suspension at a density of 40 × 10^6^ cells/ml mixed with silica microbeads was then injected into the microfluidic chamber of the AFS chip and incubated. Nonattached and dead cells were flushed out by connecting the AFS chip to a syringe pump (AL-2000, World Precision Instruments) after 2 h of incubation. HUVECs were then grown inside the incubator overnight at a slow flow rate (2 μL/min). On the day of measurement, each bead in the field of view was tracked by LabVIEW, and their z-position was determined by a look-up table (LUT), set to track from 0 to 10,000 nm at a step size of 100 nm. After calibration, a constant acoustic force (≈1.7 nN) was applied, pushing the beads upward and thereby stretching the HUVECs. All measurements were performed at 37 °C, with an amplitude of 40–60% at a 14.47 MHz frequency, applied constantly for a period of 20 s. For determining the viscoelastic properties of HUVECs after NS1 exposure, HUVECs inside the AFS chip were further cultured in the presence of 5 μg/mL of NS1 for an additional 4 h.

### Determining the Viscoelastic Properties of HUVECs after NS1 Exposure

The viscoelastic properties of HUVECs were determined by using the power law model. The creep compliance is given by
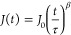
8where *J*_0_ is the material compliance, τ is the normalizing time of 1 s, and β is the power law exponent referring to the fluidity of the cells. The modulus scaling parameter, *E*_0_, was found by
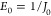
9

The extension curves (*z*-height versus time) are first converted to *J*(*t*) by
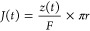
10where *z*(*t*) is the extension curve (*z*-direction) obtained by pulling on a cell, *F* is the applied force, and *r* is the radius of the silica microbead.

### Statistical Analysis

Statistical analyses and data visualization were performed using GraphPad Prism (version 9.4.0). Outliers in the box plots were identified using IBM SPSS Statistics (version 25). The plotted data represent the mean ± standard error of the mean (SEM) from three or four independent replicates. Data normality was assessed with Shapiro–Wilk test. For rheology experiments, the mixed-effects multiple comparisons with Bonferroni correction were used to compare *G*′ and *G*″ between control and NS1-exposed conditions, at each frequency. For AFS experiments, the nonparametric Mann–Whitney *U* test was used to determine significant differences between independent conditions. For permeability measurements in the organoplate, *p*-values were determined using an unpaired Student’s *t*-test and one-way analysis of variance followed by Dunnett’s multiple comparisons test, with significance levels set at *p < 0.05, **p < 0.01, ***p < 0.001, and ****p < 0.0001.

## Results

### NS1 Softens Single Endothelial Cells

**Figure 1:**
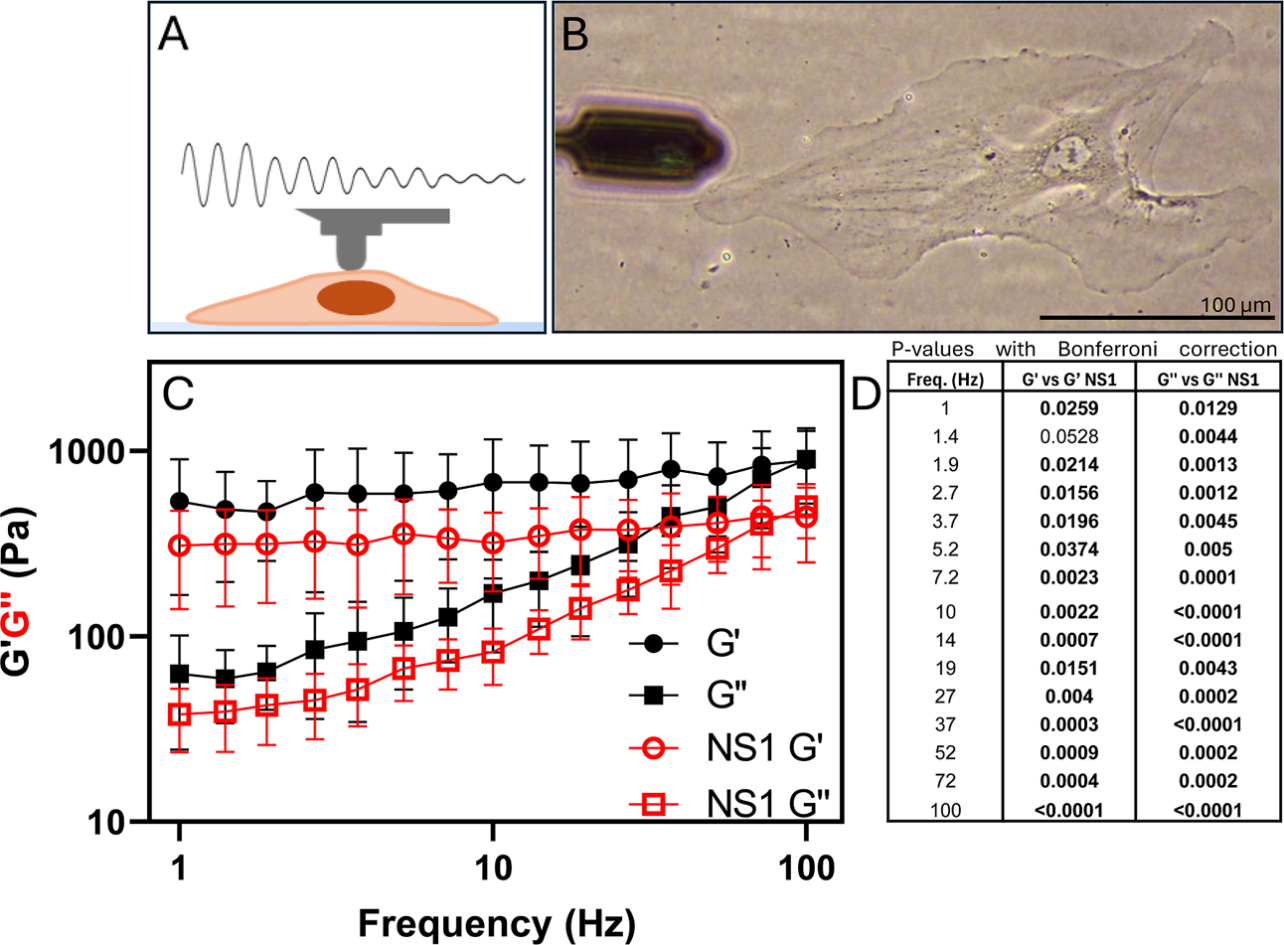
NS1 softens single endothelial cells. (A) Schematic representation of the experimental setup, illustrating the indentation of the cells by the probe. (B) Microscopic image showing a single endothelial cell and the probe. (C) Exposure to NS1 significantly lowers (*P* < 0.05) the shear storage, *G*′ and shear loss, *G*″ of endothelial cells exposed to NS1 compared to the control. (D) *P*-values calculated using mixed-effects multiple comparisons with Bonferroni correction. Comparisons include mean differences of *G*′ and *G*″ between control and NS1-exposed condition for each applied frequency. Endothelial cells were exposed to 5 μg/mL NS1 for 4 h. Data represent the mean stiffness of 10 cells per experiment, with three independent biological replicates. Error bars indicate the standard deviation across replicates.

To evaluate the impact of Dengue virus NS1 on the mechanical properties of endothelial cells, we conducted frequency-dependent rheological measurements using scanning probe microscopy (Figure 1A,B). Both the shear storage (*G*′) and shear loss (*G*″), which respectively reflect the elastic and viscous components of cellular mechanics, were assessed across a broad frequency range to characterize the viscoelastic response under control and NS1-exposed conditions.

Our single-cell rheology analysis revealed no significant qualitative differences in the frequency-dependent profiles of *G*′ and *G*″ between the two conditions, indicating that the overall viscoelastic behavior of the cells remained consistent (Figure 1C). However, absolute values of both *G*′ and *G*″ were significantly lower in cells exposed to NS1 compared to controls (*p* < 0.05). Specifically, *G*′ exhibited a pronounced decrease, indicating a reduction in the rigidity of the cells, while the concomitant reduction in *G*″ reflects an attenuated ability to dissipate mechanical energy (Figure 1C,D). These changes collectively indicate that NS1 exposure induces cellular softening.

**Figure 2:**
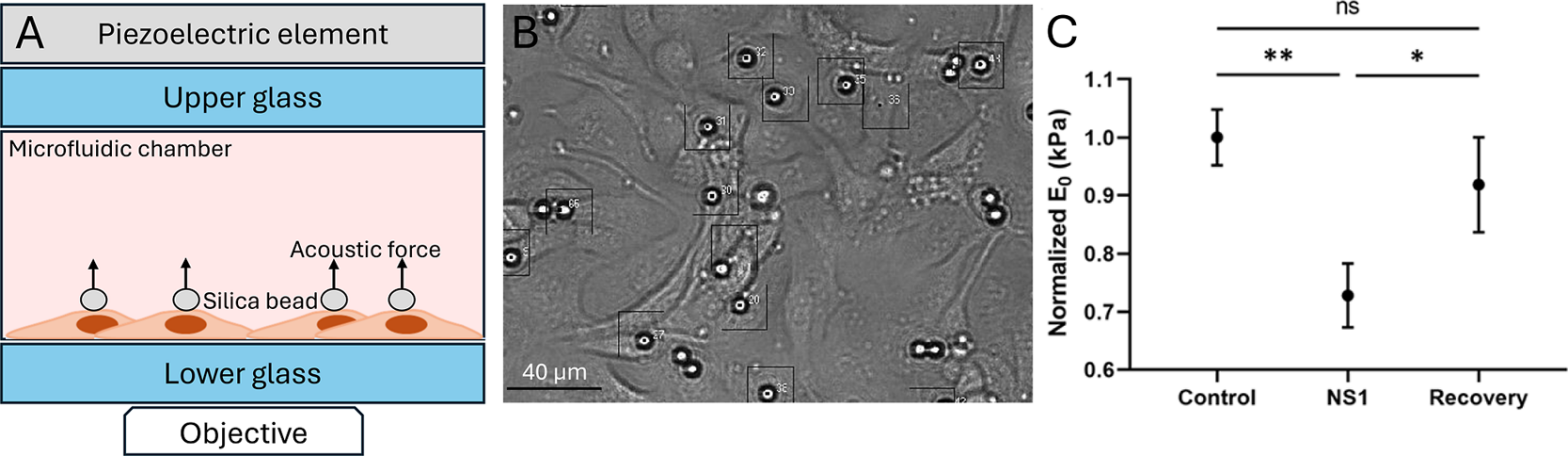
NS1 softens endothelial cells in a monolayer. (A) Schematic representation of the cross-sectional layer of the AFS microfluidic chip with a transparent piezo element. Standing acoustic wave drives silica microbeads to the acoustic node, thereby stretching the cells. (B) HUVECs with silica microbeads were cultured inside the AFS microfluidic chip for 2 h at 37 °C with 5% CO_2_. (C) Exposure to 5 μg/mL of NS1 significantly softened the HUVEC cells within 4 h. No statistically significant differences in stiffness were observed after washing out the viral proteins and culturing them overnight. All experiments were performed at 37 °C, with sample sizes of *n* = 140, 78, and 57 for control, NS1, and recovery groups, respectively.

To further investigate the effect of NS1 on endothelial mechanics, we probed the viscoelastic properties of single HUVECs in a monolayer. Due to the fact that we did not observe differences in frequency-dependent profiles using scanning probe microscopy, here, we used acoustic force spectroscopy which enables us to probe viscoelastic properties in high-throughput. In the experimental setup, HUVECs were confined between silica microbeads and the glass surface of the AFS microfluidic chip (Figure 2A,B). Acoustic forces from a piezo element generated standing waves, pushing beads toward the acoustic node and stretching the cells at a constant stress. Real-time tracking of *z*-directional bead displacement measured cell strain. Only single beads on top of cells were analyzed to avoid surface interaction effects.

Exposure to NS1 resulted in a significant reduction (*P* < 0.05) in the apparent stiffness (*E*_0_) of HUVECs, consistent with the softening observed in the scanning probe microscopy experiments (Figure 2C). Notably, this effect was reversible; stiffness recovered to baseline levels after the cells were cultured overnight following the removal of NS1. These findings highlight the transient nature of NS1-induced mechanical alterations and suggest a potential for recovery of endothelial mechanics upon cessation of viral protein exposure.

### NS1 Induces Vascular Barrier Disruption in the Microvessel-on-a-chip

**Figure 3:**
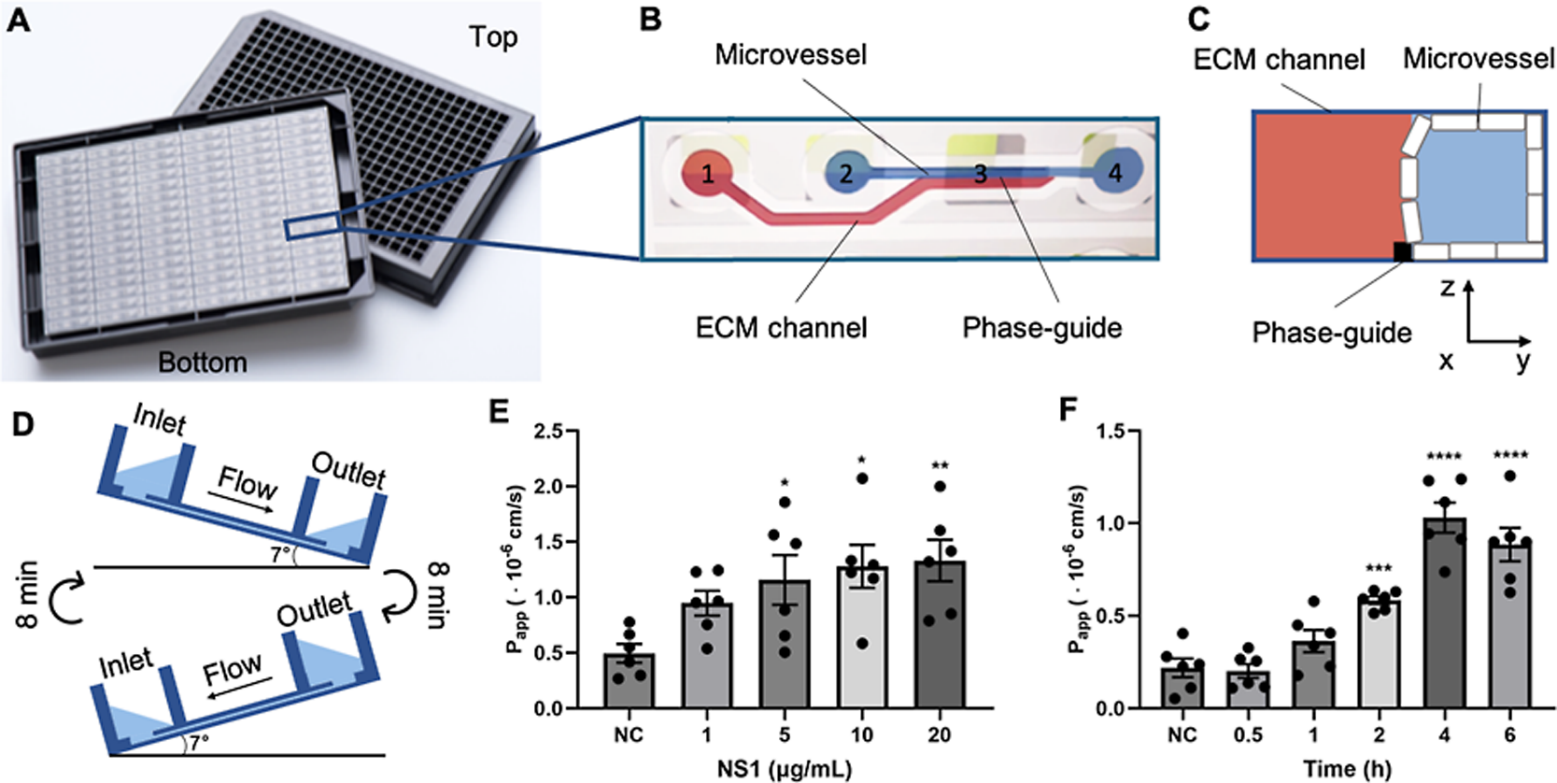
NS1 disrupts vascular barrier integrity in microvessel-on-a-chip. (A) Schematic diagram of the OrganoPlate, featuring a 384-well plate interface on top and 96 integrated microfluidic chips at the bottom. (B) Diagram of a single microfluidic chip, comprising a microvessel and an ECM channel separated by a phase-guide; 1 = gel inlet, 2 = medium inlet, 3 = observation window, 4 = medium outlet. (C) Compartments of the microvessel and ECM channel. A monolayer of HUVECs forms a microvessel adjacent to the ECM channel within the microfluidic system. (D) Perfusion in the OrganoPlate is achieved through bidirectional flow generated by a tilted rocking platform with an 8 min rocking interval. (E) Dose response of microvessels to NS1-induced apparent permeability (*P*_app_) increases after 4 h of exposure. (F) *P*_App_ of microvessels in response to 5 μg/mL NS1 at different time points (0.5, 1, 2, 4, 6 h). Data are presented as mean ± SEM; *n* = 6 (independent chips using HUVECs from 3 to 5 different donors). Panels A-D are reproduced or adapted from ref (22). Available under a CC BY license. Copyright 2021 Tang et al.

NS1 treatment is expected to affect the mechanical integrity of microvessels, leading to leakage, which can, in principle, be mimicked in an organ chip with a phase guide structure. To recapitulate and investigate the barrier integrity of the endothelium in dengue virus disease, we exposed engineered microvessels to various concentrations of NS1 (1, 5, 10, and 20 μg/mL). These microvessels were constructed using a 384-well microtiter plate (Figure 3A) equipped with microfluidic tissue chips. The phase-guide channel system in these chips allows for precise collagen patterning and HUVEC culture (Figure 3B,C), generating up to 96 perfusable microvessels. To ensure optimal HUVEC growth, the plate was placed on a rocker platform to create continuous, bidirectional flow within the vessels (Figure 3D). NS1 infusion resulted in a dramatic, dose- and time-dependent increase in the permeability of the engineered microvessels (Figure 3E,F).

### NS1 Induces Vascular Barrier Disruption through Modulating Endothelial VE-Cadherin, F-Actin, and Hyaluronan

**Figure 4:**
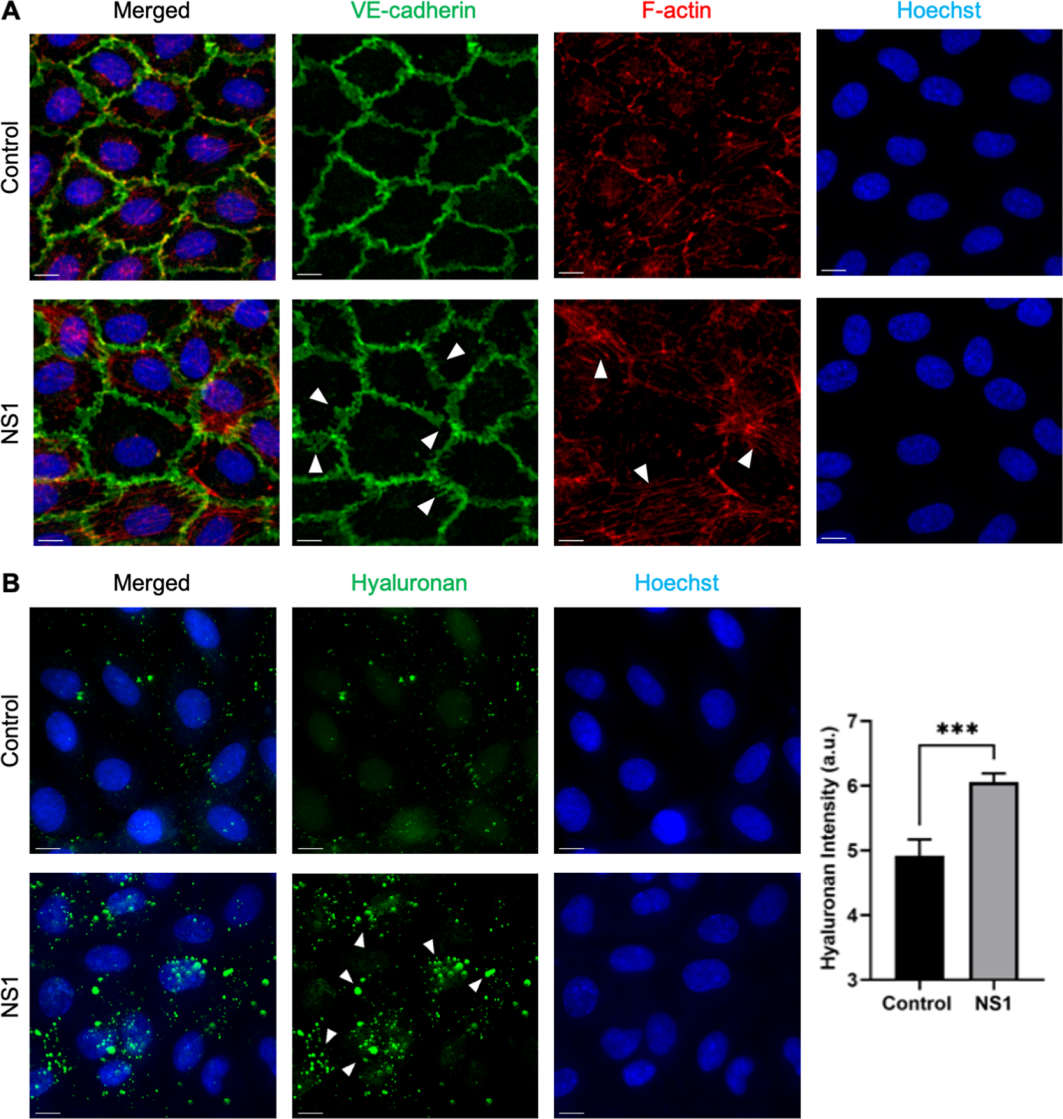
NS1-induced vascular leakage is associated with the rearrangement of VE-cadherin, formation of stress fibers, and synthesis of hyaluronan. (A) Immunostaining of endothelial cells in the microvessel-on-a-chip for VE-cadherin (green) and F-actin (red) following incubation with 5 μg/mL NS1 for 4 h. An increase in the formation of actin stress fibers and morphological rearrangement of VE-cadherin were observed (arrowheads). Scale bars: 30 μm. (B) Endothelial cells, both untreated and NS1-treated, were stained for hyaluronan (green), revealing the presence of hyaluronan-rich dot-like structures (arrowheads). Signal intensity was quantified from three independent experiments. Data are presented as mean ± SEM of 5 independent chips from 3 to 5 donors, with statistical significance indicated as ***p < 0.001. Scale bars: 30 μm.

To elucidate the potential mechanisms underlying NS1-induced disruption of the vascular barrier, we examined alterations in the expression of endothelial VE-cadherin, F-actin, and hyaluronan, three proteins crucial for endothelial mechanics. Our results demonstrate that NS1-induced vascular leakage is associated with the rearrangement of VE-cadherin, formation of stress fibers, and synthesis of hyaluronan. The disruption of the endothelial barrier, characterized by the dissociation of VE-cadherin into a zigzag pattern with visible gaps between cells, suggests the internalization of VE-cadherin (Figure 4A). Pearson correlation coefficient was lower in microvessels exposed to DENV NS1 (0.248 ± 0.02) compared to the negative control (0.363 ± 0.04), indicating a significant increase in the formation of stress fibers (*p* = 0.0017). Furthermore, enhanced synthesis of hyaluronan and the presence of hyaluronan-rich dot-like structures were observed in response to NS1 in microvascular endothelial cells (Figure 4B).

## Discussion

Vasculopathy is a critical and potentially fatal consequence of Dengue virus infection, significantly contributing to the severity of Dengue hemorrhagic fever and Dengue shock syndrome. While Dengue could be referred to as “a disease of mechanics,” the effects of the virus on the mechanical properties of endothelial cells remain poorly understood. This study addresses this knowledge gap by characterizing the mechanical alterations in the endothelium induced by Dengue virus NS1 using a combination of microvessel-on-a-chip technology and single-cell force spectroscopy. Our findings provide novel insights into the interplay between NS1 and endothelial mechanics, emphasizing its role in vascular dysfunction during Dengue infection. The work also introduces new technologies to the field, opening new avenues for fundamental and translation research.

### Mechanistic Insights into NS1-Induced Endothelial Dysfunction

Our results reveal that NS1 disrupts vascular barrier integrity through a dose- and time-dependent modulation of VE-cadherin, F-actin, and hyaluronan, leading to significant softening of endothelial cells. VE-cadherin, a key component of adherens junctions, is essential for maintaining endothelial integrity. It physically interacts with actin filaments, providing mechanical strength and stability to cell–cell junctions. Disruption of VE-cadherin localization and actin filament rearrangement, as observed in our study, increases intercellular gaps, exacerbating vascular leakage. These findings align with prior studies highlighting the role of VE-cadherin and cytoskeletal dynamics in endothelial permeability.^23,24^

In addition to junctional disruption, our data underscore the critical role of hyaluronan, a major component of the endothelial glycocalyx, in NS1-induced vascular dysfunction. Hyaluronan plays a dual role: it contributes to glycocalyx integrity under homeostatic conditions but becomes a mediator of damage when degraded by hyaluronidase or reactive oxygen species (ROS). Clinical studies have reported elevated serum hyaluronan levels in patients with severe Dengue infection, correlating with disease severity and vascular leakage.^25,26^ Our observation of increased hyaluronan production and deposition in NS1-treated endothelial cells provides a mechanistic explanation for these clinical findings, suggesting that NS1 directly modulates glycocalyx components, contributing to endothelial softening and permeability. This complements prior research indicating that Dengue NS1 induces disruption of endothelial glycocalyx components such as endothelial sialidases, syndecan-1 cathepsin L, and heparan sulfate, leading to vascular leak.^27,28^

### Reversible Softening of Endothelial Cells: Implications for Therapeutic Intervention

An intriguing finding of our study is the reversibility of endothelial softening upon removal of NS1. This observation suggests that early intervention targeting NS1 or its downstream signaling pathways could restore endothelial mechanics and prevent irreversible damage. Therapeutic strategies aimed at stabilizing VE-cadherin interactions or preventing glycocalyx degradation could hold promise in mitigating Dengue-induced vasculopathy. Recent studies have explored the use of monoclonal antibodies against NS1 to prevent its interaction with endothelial cells, thereby preserving vascular integrity.^29,30^ Our microvessel-on-a-chip model provides a robust platform to evaluate the efficacy of such therapeutic antibodies, offering a physiologically relevant context for preclinical drug screening.

### Broader Implications and Future Directions

This study underscores the mechanobiological perspective of Dengue pathology, framing the virus as a disruptor of endothelial mechanics. The “disease of mechanics” concept extends beyond Dengue, offering parallels to other viral infections characterized by vascular dysfunction, such as Zika virus and Ebola virus. Comparative studies could elucidate shared and unique mechanisms of endothelial disruption across different viral infections, potentially identifying conserved therapeutic targets. Furthermore, our organ chip model of Dengue hemorrhagic syndrome represents a significant technological advancement in disease modeling. Unlike traditional in vitro or animal models, organ-on-chip platforms recapitulate the complex microenvironment of human vasculature, providing unparalleled insights into disease mechanisms.^31^ This technology can be used not only for therapeutic screening but also for studying host–pathogen interactions and testing pharmacokinetics of potential treatments. For instance, the application of microfluidic systems to model vascular shear stress could further refine our understanding of how mechanical forces interact with NS1-mediated endothelial dysfunction.

### Clinical Relevance and Translational Potential

The translational potential of our findings lies in their ability to inform therapeutic strategies and guide clinical decision-making. Elevated serum hyaluronan levels, as reported in severe Dengue cases, could serve as a biomarker for early detection of vascular complications. Additionally, targeting the pathways involved in glycocalyx degradation and cytoskeletal reorganization may offer new therapeutic avenues. Developing a pharmacokinetic model using our organ chip platform could facilitate the design of optimal dosing regimens for existing and novel treatments, ultimately improving patient outcomes.

### Limitations

Despite the significant insights gained, our study has several limitations that warrant consideration. First, while our microvessel-on-a-chip model recapitulates key aspects of human vascular physiology, it cannot fully replicate the complexity of in vivo conditions, including interactions with immune cells and systemic inflammatory responses. Future studies integrating immune components may provide a more comprehensive understanding of NS1-induced endothelial dysfunction. Second, our findings are based on in vitro exposure to NS1 at specific concentrations and time points. The relevance of these experimental conditions to the dynamic and variable levels of NS1 during natural infection remains to be fully elucidated. Longitudinal studies measuring NS1 levels in patients and correlating them with endothelial mechanics could strengthen the translational relevance of our findings. Third, while we demonstrate that NS1 induces endothelial softening, a clear mechanistic link between NS1-induced vascular junction disruption and softening remains to be established. Although disruption of the actin or microtubule network leads to the formation of paracellular gaps and increased permeability,^32^ a more detailed analysis of the relationship between junctional integrity and mechanical properties is needed to reinforce the causal mechanisms underlying Dengue NS1-induced endothelial dysfunction. Fourth, the reversibility of endothelial softening observed upon removal of NS1 suggests potential therapeutic avenues, but the underlying signaling pathways responsible for this recovery remain unclear. Identifying these pathways will be crucial for understanding the potential for clinical intervention and therapeutic development. Finally, our study focuses primarily on the effects of NS1 on endothelial cells. Given the multifactorial nature of Dengue pathogenesis, other viral proteins and host factors likely contribute to vascular dysfunction. Expanding the scope of research to include these factors will provide a more holistic view of the disease.

## Conclusion

In conclusion, our study provides critical insights into the mechanobiological underpinnings of Dengue-induced vascular dysfunction. By elucidating the role of NS1 in modulating endothelial mechanics, we pave the way for targeted therapeutic interventions aimed at preserving vascular integrity. The integration of advanced technologies such as organ chip and lab-on-a-chip (for cellular force spectroscopy) platforms underscores the importance of innovative approaches in understanding and combating complex diseases like Dengue. Moving forward, deeper exploration of the interplay between endothelial mechanics and immune responses will be essential to fully unravel the pathophysiology of Dengue and related vascular diseases.
